# Corrigendum

**DOI:** 10.1002/ueg2.12257

**Published:** 2022-05-31

**Authors:** Takeshi Ogura

In the paper^1^ published in 2019 the authors wish to correct the proprietary name of the dilation device EZ Dilator. The name of the device should be ES Dilator.
